# Supplementary material for: Systemic Innate Immune System Restoration as a Therapeutic Approach for Neurodegenerative Disease: Effects of NP001 on Amyotrophic Lateral Sclerosis (ALS) Progression
Source: Biomedicines. 2024 Oct 16;12(10):2362. doi: 10.3390/biomedicines12102362 (PMC11505581; doi:10.3390/biomedicines12102362)
Supplement: Supplementary file 1 [file biomedicines-12-02362-s001.zip › biomedicines-3222226-supplementary.pdf]

Appendix to manuscript entitled "Systemic Innate Immune System Restoration as a Therapeutic Approach for Neurodegenerative Disease: Effects of NP001 on Amyotrophic Lateral Sclerosis (ALS) Progression"

Supplemental Table S1. Baseline demographics and characteristics of phase 2A and 2B completers [1].

Characteristics	NP001 2mg/kg (*n* = 91)	Placebo (*n* = 98)	*p* value
Sex, *n* (%)			0.53
Female	31 (34.1%)	29 (29.6%)	
Male	60 (65.9%)	69 (70.4%)	
Age at baseline, year	55.9 ± 10.8	55.4 ± 10.6	0.65
Type of ALS, *n* (%)			0.002
Familial	3 (3.3%)	17 (17.3%)	
Sporadic	88 (96.7%)	81 (82.7%)	
Site of ALS onset, *n* (%)			
Bulbar	12 (13.2%)	16 (16.3%)	0.68
Limb	79 (86.8%)	82 (83.7%)	
El Escorial criteria for ALS, *n* (%)			NS
Definite	40 (44.0%)	42 (42.9%)	
Possible	7 (7.7%)	6 (6.1%)	
Probable	39 (42.9%)	44 (44.9%)	
Probable Laboratory Supported	5 (5.5%)	6 (6.1%)	
Concurrent riluzole use, *n* (%)			1.0
Yes	66 (72.5%)	71 (72.4%)	
No	25 (27.5%)	27 (27.6%)	
ALSFRS-R score at baseline, mean ± SD	38.5 ± 4.9	37.8 ± 5.2	0.39
Vital capacity at baseline, mean ± SD	96.5 ± 20.0	93.0 ± 19.6	0.27
Months since ALS symptom onset [2], mean ± SD	18.22 ± 8.55	17.96 ± 8.13	0.81
Creatinine at baseline (μM/L), mean ± SD	64.6 ± 16.0	65.8 ± 15.4	0.52

Abbreviation: n, number of participants. NS, not significant. SD, standard deviation. [1] Completers, participants who had completed the 6-cycle treatments and had ALSFRS-R total score assessment at the end of study. [2] Months from ALS symptom onset to baseline.

Supplemental Table S2. Baseline demographics and characteristics of high creatinine group [1] in completers [2] with plasma CRP > 1.13 mg/L at baseline.

Characteristics	NP001 2mg/kg (*n* = 47)	Placebo (*n* = 51)	*p* value
Sex, *n* (%)			0.22
Female	22 (46.8%)	17 (33.3%)	
Male	25 (53.2%)	34 (66.7%)	
Age at baseline, year	58.5 ± 7.4	57.4 ± 11.2	0.97
Site of ALS onset, *n* (%)			0.20
Bulbar	6 (12.8%)	12 (23.5%)	
Limb	41 (87.2%)	39 (76.5%)	
El Escorial criteria for ALS, *n* (%)			NS
Definite	21 (44.7%)	24 (47.1%)	
Possible	5 (10.6%)	2 (3.9%)	
Probable	17 (36.2%)	22 (43.1%)	
Probable Laboratory Supported	4 (8.5%)	3 (5.9%)	
Concurrent riluzole use, *n* (%)			1.0
Yes	35 (74.5%)	39 (76.5%)	
No	12 (25.5%)	12 (23.5%)	
ALSFRS-R score at baseline, mean ± SD	39.2 ± 4.5	38.2 ± 5.1	0.32
Vital capacity at baseline, mean ± SD	90.6 ± 17.2	89.8 ± 17.89.4	0.97
Months since ALS symptom onset [3], mean ± SD	18.23 ± 8.37	16.63 ± 7.78	0.27
Creatinine at Baseline (μM/L), mean ± SD	71.0 ± 16.5	73.8 ± 11.9	0.16

Abbreviation: n, number of participants. NS, not significant. SD, standard deviation. [1] High creatine group: participants with baseline creatinine levels for males ≥ 71 μM/L & females ≥ 53 μM/L. [2] Completers: participants who had completed the 6-cycle treatments and had ALSFRS-R total score assessment at the end of study. [3] Months from ALS symptom onset to baseline.

Supplement Figure 1.

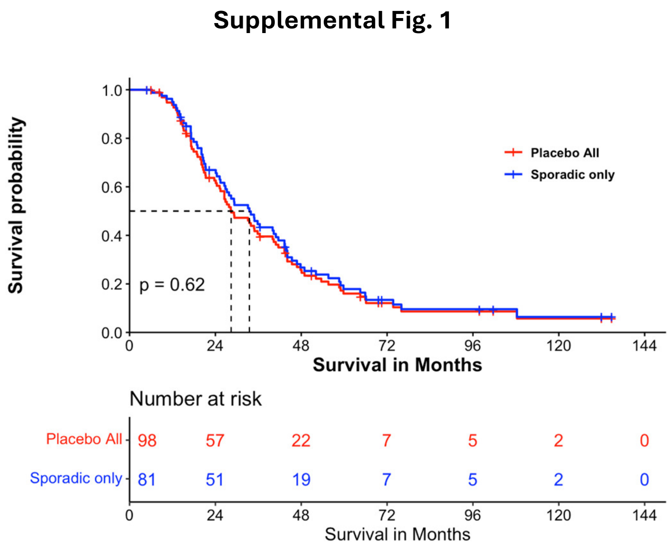

Supplemental Figure S1. Overall survival in placebos with or without cases of familial ALS in phase 2A and 2B completers. Kaplan-Meier curve of survival probability for the entire placebo group (sporadic + familial, red) compared with placebos without familial (sporadic only, blue) in the completers. The median survival (95% confidence interval [CI]) over the entire follow-up duration among placebos with familial was 28.4 months (95% CI: 25.4, 40.0) and 33.5 months (95% CI: 26.8, 43.3) in placebos without familial, respectively (log-rank, $p = 0.62$). The associated hazard ratio (HR) was 0.92 (95% CI: 0.67, 1.3) for placebos without familial vs all placebos.
